# Mutation accumulation and horizontal gene transfer in *Escherichia coli* colonizing the gut of old mice

**DOI:** 10.1080/19420889.2020.1783059

**Published:** 2020-06-24

**Authors:** Hugo C. Barreto, Nelson Frazão, Ana Sousa, Anke Konrad, Isabel Gordo

**Affiliations:** a Instituto Gulbenkian de Ciência, Oeiras, Portugal; b IBiMed, Institute for Biomedicine, Universidade de Aveiro, Aveiro, Portugal

**Keywords:** experimental evolution, aging, mutation, horizontal gene transfer, *Escherichia coli*

## Abstract

The ecology and environment of the microbes that inhabit the mammalian intestine undergoes several changes as the host ages. Here, we ask if the selection pressure experienced by a new strain colonizing the aging gut differs from that in the gut of young adults. Using experimental evolution in mice after a short antibiotic treatment, as a model for a common clinical situation, we show that a new colonizing *E. coli* strain rapidly adapts to the aging gut via both mutation accumulation and bacteriophage-mediated horizontal gene transfer (HGT). The pattern of evolution of *E. coli* in aging mice is characterized by a larger number of transposable element insertions and intergenic mutations compared to that in young mice, which is consistent with the gut of aging hosts harboring a stressful and iron limiting environment.

## Introduction

The passage of time in the life of organisms is accompanied by a myriad of changes that ultimately culminate in death. In his extraordinary comparative physiology study “The prolongation of life: Optimistic studies”[1], Metchnikoff postulated that the intestine is the organ to blame for short lifespans; with time it is increasingly colonized by harmful bacteria instead of beneficial ones. Today’s sequencing technologies, which permit the cataloging of many bacterial species in the intestines of hosts, indeed reveal significant compositional changes of gut bacterial species as the host clock ticks and its intestine ages [2]. However, another clock accompanies the ecological changes in the gut: the evolutionary time taken by each bacterial species to produce new strains [3]. Recently, we observed that under continuous antibiotic treatment, the common commensal *Escherichia coli* evolves differently in the gut of old mice relative to genetically similar but younger mice: shifts from metabolic adaptations to mutations targeting stress-related functions [4] were observed. As continuous antibiotic treatment can strongly affect the gut microbial ecosystem as well as host physiology [5], we now focus on the differences in evolutionary responses of this important human commensal after treatment is stopped in old and young mice.

## Results and discussion

We colonized a cohort of old mice (18.5 months, n = 9) after a short treatment with streptomycin [6], with an invader *E. coli* strain, labeled with two neutral markers coding for either YFP or CFP fluorescence. These markers allow rapid detection of evolutionary adaptation to the gut through the emergence and spread of new mutations within the focal newly colonizing strains. The short treatment with streptomycin is able to break colonization resistance, allowing the invader *E. coli* to colonize the mouse gut under a complex gut microbiota, which may harbor a resident *E. coli* strain [6] and can affect its evolutionary trajectories [7].

**Figure 1.**
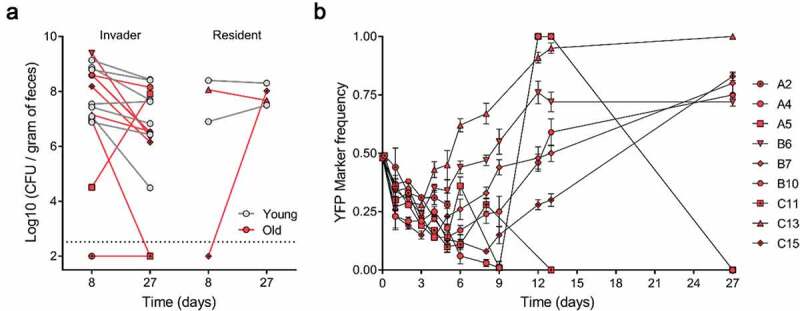
Abundances and adaptive evolution of colonizing *E. coli*. (a) Bacterial loads per gram of feces of the invader and resident *E. coli* in old (red symbol, n = 9) and in young mice (gray symbol, n = 7). Each symbol represents the average bacterial loads per gram of feces of each mouse (at least 3 measurements); (b) Temporal dynamics of the frequency of the YFP-labeled *E. coli* populations evolving in old animals (n = 9). The error bars represent the ±2*SEM.

To characterize differences in the colonization of the invader *E. coli* in the gut microbiota of young and old mice we estimated its total loads. The invader *E. coli* can stably colonize the majority of old mice with loads similar to those in young mice [6] and is also able to colonize in the presence of a resident *E. coli* that is native to the mouse microbiota (mice C13 and C15) (Figure 1a) leading to strain co-existence. No differences in the mean abundances for the invader strain of *E. coli* were found between old and young mice (One-way ANOVA with Bonferroni’s multiple comparison test for Log_10_(CFU/gram of feces), p > 0.05), both at d 8 and 27 post-colonization (Figure 1a). We were not able to detect *E. coli* in the old mouse A2 after 8 d of colonization and in the old mouse A2 and C11 after 27 d of colonization (Figure 1a).

To query the speed of evolution during the 27 d of colonization, we measured the changes in the frequency of the neutral markers within the invader strain. We observed that YFP frequency changes rapidly, with increases followed by declines over time (Figure 1b), a pattern that is expected under rapid evolution and intense clonal interference [4].

**Figure 2.**
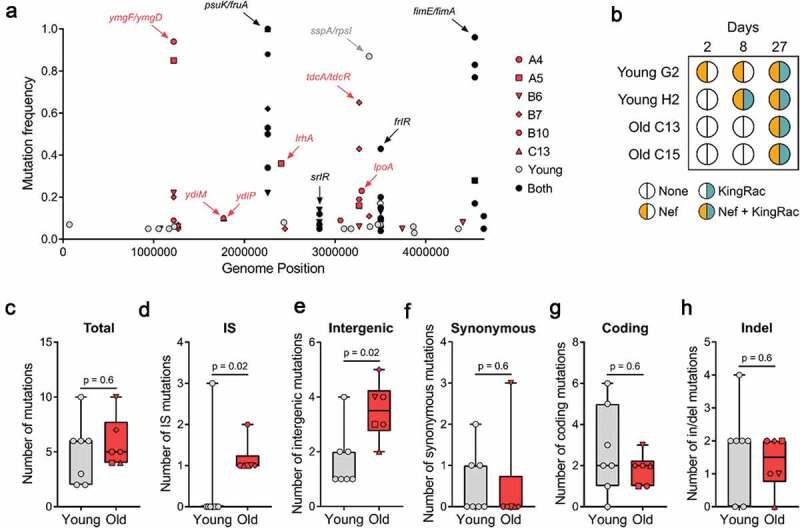
Mutation accumulation and Horizontal Gene Transfer in *E. coli*. (a) Frequency of the mutations specific to the young (n = 7) in gray, old (n = 6, mouse A4, A5, B6, B7, B10, C13) in red, and common to both age groups in black; (b) Bacteriophage-mediated HGT detected in young (n = 2) and old (n = 2) mice. Nef and KingRac bacteriophages were typed in 20 clones at d 2 and 8 and in a pool of >1000 clones at d 27; (c) Total number of mutations, (d) IS mutations, (e) intergenic mutations, (f) synonymous mutations, (g) coding mutations, and (h) insertion/deletion mutations detected in *E. coli* populations evolved in young (n = 7) and old (n = 6) mice 27 d post-colonization. For all boxplots, the line inside the box indicates the median, the lower and upper box boundaries indicate the 25th and 75th percentile, respectively, and the whiskers range from the minimum to the maximum value.

To determine the pattern of evolution and identify the mutational targets of adaptation to the gut of old mice, we performed whole-genome sequencing of the invader *E. coli* populations after 27 d of colonization. Old mice A2 and C11 were excluded from this analysis due to the low loads at d 27 as well as C13 due to the emergence of streptomycin-resistant bacteria. We find that evolution in the gut of old mice involves accumulation of mutations in *frlR*, in *srlR*, in the intergenic region *psuK/fruA*, and in the intergenic region of *fimE/fimA*, all of which were previously observed when this strain colonized young mice [3,6,8,9] (Figure 2a, Tables 1 and 2). Mutations in these targets are likely adaptive in both age groups as they emerge in multiple mice independently and/or with multiple alleles within the same mouse (e.g. *srlR* mutation is detected only in mouse B6 of the old age cohort but with mutations leading to two distinct amino-acid changes). Both, *frlR* (putative transcriptional regulator of the fructoselysine operon [10]) *and psuK/fruA* (*psuK* is involved in pyrimidine metabolism [11] and *fruA* in fructose transport [12]), as well as *srlR* (repressor of sorbitol transport and utilization [13]), may be related to the mouse diet, as they are also observed when *E. coli* colonizes germ-free mice [7]. FimE is known to regulate *fimA*, which is the major subunit of type 1 fimbriae. Importantly we find mutations in the evolving invader *E. coli* that are specific to old mice. Some of these reached considerable frequencies, such as mutations in *lrhA, lpoA, ydiM*, and *ydiP* (Figure 2a). Mutations in *lrhA* were previously observed in old mice undergoing continuous antibiotic treatment and shown to increase *E. coli* motility [4], suggesting that their selection is independent of the antibiotic. Interestingly, LrhA is also known to regulate *fimE* [14]. LpoA plays a critical role in the *in vivo* function of penicillin-binding protein A [15] and *ydiM* is involved in isoprenol tolerance [16]. A remarkable signal of convergent evolution in the cohort of old mice was detected in the intergenic regions *ymgF/ymgD* and *tdcA/tdcR* (Figure 2a). The latter genes are involved in the anaerobic degradation of L-threonine to propionate [17], associated with stress response in *Klebsiella pneumoniae* [18], and iron limitation in a pathogenic strain of *E. coli* [19]. We observed IS5 insertions in the intergenic region *tdcA/tdcR* reaching frequencies as high as 65% in one mouse and above 5% in 4 out of 6 mice (Figure 2a, Table 1), suggesting that this region is under strong selection in this environment.

**Table 1.** Evolutionary changes detected in the invader *Escherichia coli* after colonization of old mice for 27 d.

Mouse	Genome Position	Mutation or Prophage	Gene	Annotation	Frequency
A4	1219708	+1 T	*ymgF→/ymgD←*	Intergenic	0.94
	3293090	C *→* T	*lpoA*	R *→* C	0.23
	4540742	Δ 1 bp	***fimE→/fimA→***	Intergenic	0.77
	4540714-4541009	Inversion 296 bp	***fimE→/fimA→***	Intergenic	0.17
	4638770	IS5	***yjjY→/yjtD→***	Intergenic	0.11
A5	1219708	+1 T	*ymgF→/ymgD←*	Intergenic	0.85
	2404622	+6TCGAGG	*lrhA*	Codon insertion	0.36
	3265168	IS5	*tdcA←/tdcR→*	Intergenic	0.16
	4540714-4541009	Inversion 296 bp	***fimE→/fimA→***	Intergenic	0.28
B6	1072335	G *→* T	*rutA*	Synonymous	0.05
	1219708	+1 T	*ymgF→/ymgD←*	Intergenic	0.22
	1269768	G *→* T	*ldrC←/chaA←*	Intergenic	0.06
	2257444	A *→* C	***psuK←/fruA←***	Intergenic	0.22
	2827271	A *→* T	***srlR***	D *→* V	0.08
	2827492	G *→* A	***srlR***	G *→* S	0.14
	3265168	IS5	*tdcA←/tdcR→*	Intergenic	0.06
	3502132	T *→* A	***frlR***	L *→* Q	0.13
	3741217	A *→* C	*yiaK*	Synonymous	0.05
	4409190	G *→* A	*yjfJ*	Synonymous	0.08
B7	1219708	+1 T	*ymgF→/ymgD←*	Intergenic	0.20
	1269768	G *→* T	*ldrC←/chaA←*	Intergenic	0.05
	2257444	A *→* C	***psuK←/fruA←***	Intergenic	0.62
	2447106	Δ 10 bp	*smrB*	Frameshift	0.05
	3265132	A *→* C	*tdcA←/tdcR→*	Intergenic	0.43
	3265168	IS5	*tdcA←/tdcR→*	Intergenic	0.65
	3375262	T *→* G	*sspA*	T *→* A	0.11
B10	1219708	+1 T	*ymgF→/ymgD←*	Intergenic	0.09
	1770086	IS5	*ydiM*	Gene interrupted	0.10
	2257444	A *→* C	***psuK←/fruA←***	Intergenic	0.88
	3057781	C *→* T	*argP*	R *→* C	0.09
	3265168	IS5	*tdcA←/tdcR→*	Intergenic	0.19
C13	1269768	G *→* T	*ldrC←/chaA←*	Intergenic	0.07
	1770086	IS5	*ydiM*	Gene interrupted	0.10
	1776568	C *→* T	*ydiP*	W *→* L	0.10
	2257444	A *→* C	***psuK←/fruA←***	Intergenic	1.00
	1127918 - 1174087	Nef		Prophage	0.50
	2497344 - 2544177	KingRac		Prophage	0.50

The symbol Δ means a deletion event and a + symbol represents an insertion of the nucleotide that follows the symbol. The initials IS denote the abbreviation of insertion sequence element at the indicated position. Highlighted in bold are the target genes that are common to both *E.* *coli* evolving in young and old mice.

**Table 2.** Evolutionary changes detected in the invader *Escherichia coli* colonizing young mice. The adaptive mutations observed across independently evolved population of *E. coli* after 27 d of streptomycin in young mice [6] (see also materials and methods).

Mouse	Genome Position	Mutation or Prophage	Gene	Annotation	Frequency
A2	70512	+1 A	*araC*	Frameshift	0.07
	2827142	T → C	***srlR***	L → S	0.05
	4540742	Δ 1 bp	***fimE→/fimA→***	Intergenic	0.83
B2	939933	Δ 220 bp	*serS*	Partial gene deletion	0.05
	1168829	IS2	*ycfS*	Gene interrupted	0.05
	2827246	A → T	***srlR***	K → *	0.07
	3098844	Δ 9 bp	*ansB←/yggN←*	Intergenic	0.05
	3174841	Δ 12 bp	*cpdA*	Codons deleted	0.05
	3375452	C → T	*sspA←/rpsl←*	Intergenic	0.87
	3471634	A → C	*rpsG*	L → S	0.07
	4359283	IS2	*cadC*	Gene interrupted	0.05
	4540742	Δ 1 bp	***fimE→/fimA→***	Intergenic	0.96
	4638629	IS2	***yjjY←/yjtD→***	Intergenic	0.05
D2	2257444	A → C	***psuK←/fruA←***	Intergenic	0.50
	3873062	G → A	*dgoR*	S → *	0.03
E2	1225143	G → C	*minC*	Synonymous	0.06
	2257444	A → C	***psuK←/fruA←***	Intergenic	0.53
	2435373	G → T	*pdxB*	Synonymous	0.08
	2827697	Δ 1 bp	***srlR***	Frameshift	0.12
	3383867	Δ 1 bp	*yhcN→/yhcO←*	Intergenic	0.06
	3502208	T → A	***frlR***	Y → *	0.20
G2	3497820	Δ 1 bp	*yhfL→/frlA→*	Intergenic	0.07
	3497842	G → T	*yhfL→/frlA→*	Intergenic	0.17
	3502108	T → C	***frlR***	L → P	0.43
	3502410	Δ 1 bp	***frlR***	Frameshift	0.15
	3502675	G → T	***frlR***	C → F	0.04
	3862987	A → T	*yidE*	Synonymous	0.06
	1127918 - 1174087	Nef		Prophage	0.10
	2497344 - 2544177	KingRac		Prophage	0.00
	positions above	Nef + KingRac		Prophage	0.90
H2	1054715	T → G	*torS*	Synonymous	0.05
	2257444	A → C	***psuK←/fruA←***	Intergenic	1.00
		Nef + KingRac		Prophage	
I2	2257444	A → C	***psuK←/fruA←***	Intergenic	0.34
	3502108	T → C	***frlR***	L → P	0.10
	3502410	Δ 1 bp	***frlR***	Frameshift	0.10
	3502753	T → A	***frlR***	L → H	0.09
	3502339	Δ 1 bp	***frlR***	Frameshift	0.09
	3502519	T → G	***frlR***	I → S	0.06

Highlighted in bold are the target genes that are common to both *E. coli* evolving in young and old mice. See Table 1 legend for further details.

HGT occurs when this invader *E. coli* co-exists with the mouse resident *E. coli* [6]. In addition, environmental stress (e.g. inflammation) is known to influence the rate of HGT events [20,21]. Thus, we tested for this evolutionary mechanism by enquiring if clones sampled from mice where coexistence was seen, had acquired two known active prophages. Probing for the Nef and KingRac prophages over time in mouse C13 and C15 revealed that similar to the pattern in young mice [6], HGT occurs in the old mice as well (Figure 2b). Interestingly, the acquisition of the Nef prophage by the invader *E. coli* is detected earlier in the young mice relative to old mice (Figure 2b), suggesting that Nef phage integration occurs at a higher rate in young mice. Nef is known to provide a metabolic advantage for *E. coli* when colonizing young mice [6]. Since young and old mice differ in the concentration of a variety of gut metabolites [22], a smaller advantage of acquiring Nef could be a possible explanation for what we observe in the old mice. Another possibility is that in a more stressful environment, with high inflammation levels [20], as in the gut of old mice, the Nef prophage could be more frequently induced, which leads to bacterial death and thus less bacteria carrying Nef prophage.

As the gut of old mice shows higher levels of inflammation [4] and iron deficiency [23], *E. coli* evolving in the aging gut could exhibit a higher mutation rate and/or an altered spectrum of emerging mutations [24]. The mean number of mutations detected across the aging mice was not significantly higher than that of the young mice (5.8 ± 0.9 for old and 5.0 ± 1.1 for young mice, two-sided Mann–Whitney *U* = 17.5, *p* = 0.6; Figure 2c), suggesting that the rate of mutation accumulation is not significantly increased in the old, at least during the first month of colonization. However, the number of IS insertions that increased in frequency in the old mice was significantly higher than that observed in young mice (1.2 ± 0.2 for old and 0.4 ± 0.4 for young mice, two-sided Mann–Whitney *U* = 6.0, *p* = 0.02; Figure 2d), indicating that either the rate of spontaneous IS insertions or its adaptive value is higher in the aging intestine. The number of mutations in intergenic regions was also significantly higher in the aging gut (3.5 ± 0.4 for old and 1.7 ± 0.4 for young mice, two-sided Mann–Whitney *U* = 5.0, *p* = 0.02; Figure 2e), while the number of synonymous mutations (0.5 ± 0.5 for old and 0.6 ± 0.3 for young mice, two-sided Mann–Whitney *U* = 17.0, *p* = 0.6; figure 2f), coding mutations (1.8 ± 0.3 for old and 2.7 ± 0.8 for young mice, two-sided Mann–Whitney *U* = 16.5, *p* = 0.6; Figure 2g) and insertion/deletion mutations (1.3 ± 0.3 for old and 1.7 ± 0.5 for young mice, two-sided Mann–Whitney *U* = 17.0, *p* = 0.6; Figure 2h) was not significantly different. Another signature in the pattern of molecular evolution being driven by positive selection is the high rate of coding to synonymous mutations of 3.67 and 4.75 across the cohort of old and young mice, respectively (assuming 15 generations per day).

The targets of evolution in old mice found here are similar to the pattern previously observed when old mice underwent continuous antibiotic treatment and are compatible with environmental stress and iron scarcity [4]. Since iron is essential for both, bacterial proliferation and correct functioning of the host immune system, strict regulation of iron availability in the gut is key to prevent and control the proliferation of potential bacterial pathogens [25]. With the imbalance of iron content that emerges during aging, in conjunction with less effective immune surveillance [26], the host selective pressure against the emergence of potential pathogens should in principle be reduced. On the other hand, environmental stress (e.g. iron limitation and inflammation) may lead to an increased selective pressure for bacteria functions with pathogenic potential [4]. Interestingly, it is known that *E. coli* grown under iron limitation shows a mutational profile characterized by an eightfold increase in the rate of IS transpositions [24]. In addition, intergenic mutations are of major importance for *Pseudomonas aeruginosa* adaptation within host in cystic fibrosis patients, characterized by increased inflammation in the lungs [27]. Therefore, the significant increase of IS transpositions and intergenic mutations in the aging mouse gut may be caused by differences in the iron availability and inflammation in the elderly.

## Conclusion

We have described the pattern of evolution of an invader commensal *E. coli* colonizing the gut of old mice in comparison with that observed in young adult mice. We showed that rapid adaptation and bacteriophage-mediated HGT occur in both old and young mice, but a higher number of IS transpositions and intergenic mutations is observed in old mice, consistent with higher stress and iron limitation in the aging gut.

## Materials and methods

### In vivo *evolution experiment*

Non-littermate female mice (C57BL/6 J), aged 18.5-months (n = 9), were treated for 24 h with streptomycin (5 g L^−1^) before being orally gavaged with 100 μL of a 10^8^suspension of a 1:1 mixture of the *E. coli* strains [6] (Ancestral invader-YFP/Ancestral invader-CFP). Mice were kept in individually ventilated cages under specified pathogen-free (SPF) barrier conditions at the Animal Facility of the Instituto Gulbenkian de Ciência (IGC) with access to food and water *ad libitum*. This research project was ethically reviewed and approved by both the Ethics Committee and the Animal Welfare Body of the IGC (license reference: A009.2018), and the Portuguese National Entity that regulates the use of laboratory animals (DGAV – Direção Geral de Alimentação e Veterinária (license reference: 009676)). All experiments conducted on animals followed Portuguese (Decreto-Lei nº 113/2013) and European (Directive 2010/63/EU) legislation, concerning housing, husbandry, and animal welfare. Fecal pellets were collected over time and stored in 15% glycerol at −80°C for later analysis. Lysogeny-Broth (LB, Lennox formulation) supplemented with streptomycin (100 μg mL^−1^) and MacConkey + 0.4% Lactose was used to estimate the total loads of Ancestral invader-YFP/Ancestral invader-CFP and Resident *E. coli*, respectively. The frequency of YFP and CFP bacteria was assessed using a fluorescent stereoscope (Zeiss Stereo Lumar V12).

### Whole-genome sequencing for mutation detection

Frozen fecal pellets were serially diluted and plated on LB agar plates supplemented with streptomycin (100 μg mL^−1^). After overnight incubation at 37°C, a mixture of >1000 clones was scraped from the plates and resuspended in 1X PBS. DNA was extracted with phenol-chloroform [28]. The library construction (Pico Nextera) and sequencing were performed at the IGC Genomics facility using an Illumina MiSeq Benchtop Sequencer and an Illumina NextSeq 500. Each sample was pair-end sequenced and standard procedures produced datasets of paired-end 250 bp read pairs. Sequencing adapters were autodetected and removed using fastp [29]. Raw reads were trimmed from both sides, using window sizes of 4 base pairs across which the average base quality had to reach a minimum value of 20 to be retained. Trimmed reads were retained if they reached a minimum length of 100 bps and consisted of at least 50% base pairs which had phred scores of at least 20. The filtered fastq files were passed through the bbsplit utility of bbmap [30] with default parameters in order to resolve potential contamination issues between the invader and resident strains of *E. coli* coexisting in the gut, as well as other species from within the microbiome. Reads matching neither of the two *E. coli* strains were considered contaminating reads and stored in separate files. The fully assembled invader and resident (Accession Numbers SAMN15163769 and SAMN15163749, respectively) *E. coli* strains (previously discussed in Frazão et al. [6]) were used for this step. Plasmid sequences from the resident *E. coli* strain were used as additional potential sources of reads. A total of four file-pairs were generated from the original fastq input files in this step: one for reads best matching the invader reference, one for reads matching the resident, one for plasmid reads, and one pair containing all read pairs not properly matching either of the above categories. The mean coverage per invader *E. coli* population after the filtering amounted to 261x for populations evolved in young mice, and 413x for populations evolved in old mice. The reads sorting into invader and resident reference genomes were separately aligned to either the *E. coli* strain K-12 (substrain MG1655; Accession Number: NC_000913.2) reference or the newly assembled reference genome of the resident *E. coli*, respectively. Three separate alignment algorithms were used: BWA-sampe with default parameters [31], MOSAIK with default parameters [32], and Breseq/Bowtie [33,34]. Breseq provides both, alignment and variant analysis, and was run in polymorphism mode requiring a minimum coverage of five reads per position, a variant frequency of at least 0.05, and the absence of significant strand bias. Additional variant calling approaches were employed on top of all three alignment methodologies to identify potential additional variation in the evolved genomes, and to verify the variants called by Breseq. For SNP and indel identification, a naïve pipeline using the mpileup utility within samtools [35] and a custom filter script written in python was employed. This script filtered base calls to ensure a minimum read mapping quality of 20 and a base call quality of at least 30 for variant calling. Among these high-quality positions, initially, at least 3% of reads and a minimum of five quality reads had to support a putative SNP or indel on both strands with a strand bias (pos. strand/neg. strand) above 0.2 and below 5.0 for this mutation to be considered further. Mutations were only retained if they did not fall within repeat regions or regions of sequence breakpoints (in which case clustering of false-positive SNPs was observed). The final list of mutations consists of those variants that were identified in more than one of the alignment approaches and reached an average frequency of at least 5% between the three alignments. Putative novel insertion sites of known mobile elements, 54 known bacterial insertion sequences (https://ecoliwiki.org/colipedia/images/a/a1/All_IS_seqs_fasta.txt), 9 known representative transposons (AY485150.1, D16449.1, X61367.1, AF071413.3, V00613.1, U00004.1, AF457211.1, V00622.1, AF162223.1), and 11 phages identified in the resident *E. coli* genome using Phaster [36] (Table 3), were inferred using ISMapper [37] and panISa [38], and compared to previous predictions from Breseq. Any insertion elements movement predicted by at least two of these approaches was considered well supported. All variants identified were manually verified in IGV [39,40].

**Table 3.** Putative phages predicted within the assembled genome of the resident *E. coli*. Predictions were attained using Phaster.

	Start	End				
Phage	(In Resident Genome)		Length	Completeness	Keywords	Notes
1	1314728	1394436	79.7Kb	Intact	Integrase, lysis, lysin, terminase, head, portal, capsid, tail	*Nef
2	1809906	1835390	25.4Kb	Incomplete	Terminase, integrase	
3	2005260	2015055	9.7Kb	Incomplete	Transposase	
4	2086138	2130245	44.1Kb	Intact	Transposase, lysin, terminase, portal, head, capsid, tail	
5	2182740	2223082	40.3Kb	Intact	Tail, plate, lysin, head, terminase, capsid, portal, recombinase, integrase	
6	2286353	2333386	47.0Kb	Intact	Integrase, lysin, lysis, terminase, portal, head, tail, plate	
7	2611252	2651174	39.9Kb	Intact	Lysin, tail, terminase, portal, protease, transposase	
8	2849919	2909216	59.2Kb	Intact	Tail, head, portal, terminase, lysis, lysin, integrase	*KingRac
9	3492665	3543269	50.6Kb	Intact	Tail, plate, capsid, head, portal, transposase, terminase, lysin, recombinase, integrase	
10	4655459	4674687	19.2Kb	Incomplete	Integrase, head, transposase	
11	5206358	5241330	34.9Kb	Questionable	Tail	

### Detection of bacteriophage-mediated HGT events

Prophage (Nef or KingRac)-specific genes were PCR-amplified from clones or populations isolated at different time points as described in Frazão et al. [6].

### Statistical analysis

All statistical analyses were conducted in GraphPad Prism (version 7.04). Detailed statistics for all the experiments can be found in the figure legends and/or in the manuscript together with the n and definitions of center and dispersion. In all figures, *n* represents the number of animals that were used. Statistical significance was defined for p < 0.05 in all comparisons and calculated as described in the manuscript and/or figure legends.

## Data deposition

Genome sequencing data have been deposited with links to BioProject accession number PRJNA633524 and BioProject accession number PRJNA549246 in the NCBI BioProject database (https://www.ncbi.nlm.nih.gov/bioproject/).
